# Social factors are associated with disparities in epidemiological and operational indicators of COVID-19 surveillance in a region of the Brazilian Amazon

**DOI:** 10.3389/fpubh.2025.1662081

**Published:** 2026-03-11

**Authors:** Juliane Lima Alencar, Marina Pereira Queiroz dos Santos, Ana Lúcia da Silva Ferreira, Marcos Jessé Abrahão Silva, Diana da Costa Lobato, Joyce dos Santos Freitas, Mayara Annanda Oliveira Neves Kimura, Ricardo José de Paula Souza e Guimarães, Yan Corrêa Rodrigues, Daniele Melo Sardinha, Karla Valéria Batista Lima

**Affiliations:** 1 Programa de Pós-Graduação em Epidemiologia e Vigilância em Saúde, Instituto Evandro Chagas (PPGEVS/IEC), Ananindeua, Pará, Brazil; 2 Programa de Pós-Graduação em Biologia Parasitária na Amazônia, Universidade do Estado do Pará e Instituto Evandro Chagas (PPGBPA/UEPA/IEC), Belém, Pará, Brazil; 3 Instituto de Ciências da Saúde, Faculdade de Enfermagem, Universidade Federal do Pará (ICS/FAENF/UFPA), Belém, Pará, Brazil; 4 Programa de Pós-Graduação em Virologia, Instituto Evandro Chagas (PPGV/IEC), Ananindeua, Pará, Brazil; 5 Laboratório de Microbiologia, Unidade Acadêmica Serra Talhada (UAST), Universidade Federal Rural de Pernambuco (UFRPE), Ananindeua, Pará, Brazil

**Keywords:** COVID-19, disparities, epidemiology, health region, health surveillance, indicators

## Abstract

**Introduction:**

COVID-19 has caused substantial impacts on health, the economy, education, and quality of life worldwide, and pandemic control measures are directly associated with the quality of the pandemic response, which is essential for developing more assertive interventions for health promotion, treatment, and control of COVID-19.

**Objective:**

To evaluate epidemiological and operational indicators of COVID-19 surveillance in hospitalized patients in the state of Pará in 2021 by health mesoregion.

**Methodology:**

A cross-sectional, analytical, and ecological epidemiological study. Data were obtained from the Epidemiological Surveillance of Severe Acute Respiratory Syndrome through the Influenza Epidemiological Surveillance System (SIVEP-Gripe).

**Results:**

A total of 18,007 severe acute respiratory syndrome surveillance reports were included. In terms of incidence, there was a significant difference in the Lower Amazon, Southwest Pará, and Southeast Pará (*p* < 0.001). In terms of lethality, the highest rates were in the Lower Amazon and Metropolitan Region of Belém (*p* < 0.001). In terms of mortality, significance was observed in the Lower Amazon, the Metropolitan Region of Belém, and Southwest Pará (*p* < 0.001). For timely notification (80%), sample collection (80%), completion of the collection date (100%), recording of the molecular test date (100%), and Intensive Care Unit (ICU) admission (100%), none of the mesoregions reached the target. The criterion of confirmation, evolution, and date of evolution did not reach the target (100%) in any mesoregion. For timely case closure (80%), five mesoregions reached the goal: Lower Amazon, Marajó, Metropolitan Region of Belém, Northeast Pará, and Southeast Pará.

**Conclusion:**

Differences were observed between health mesoregions in both epidemiological and operational indicators. The most affected mesoregions were the Lower Amazon, Southwest Pará, and Southeast Pará, which have high social vulnerability and are farther from the metropolitan area of Belém, where health services with better hospital and laboratory structures are concentrated.

## Introduction

COVID-19 has caused and continues to cause substantial impacts on health, the economy, education, and quality of life worldwide. It has exposed disparities between developed and developing countries, as pandemic control actions are directly associated with the quality of epidemiological surveillance, particularly the availability of molecular diagnosis, together with other measures such as social distancing, isolation of the infected individuals, and evaluation of contact networks, which characterize active surveillance and are crucial for effective pandemic control (1–4).

On 23 October 2021, Brazil had 21,723,559 confirmed cases, 605,457 deaths, a case fatality rate of 2.8%, and a mortality rate of 288.1 per 100,000 inhabitants. In the North region, on the same date, there were 1,859,549 cases and 46,773 deaths. Within this region, the state of Pará reported the highest numbers, with 596,977 cases and 16,730 deaths (5).

In an analysis by macroregion of the state of Pará, macroregion III had a lethality rate of 2.2%, corresponding to the Lower Amazon and Tapajós regions; however, it had the highest mortality rate in the state (307.6 per 100,000 inhabitants). Macroregion I, corresponding to the metropolitan region and Marajó, had a lethality rate of 2.9%, higher than the national lethality rate of 2.0%. In contrast, macroregion IV, comprising Araguaia, Lago de Tucuruí, and Carajás, had the lowest lethality rate (1.4%). When incidence was evaluated, the entire state exceeded 7,000 per 100,000 inhabitants, although this remained lower than the national incidence of 16,535 per 100,000 inhabitants in Brazil (6).

In the studies by Sardinha et al. (7), who carried out an epidemiological analysis at the beginning of the pandemic in the state of Pará, it was shown that the health mesoregions already had disparities in incidence, with the highest rates in the south of the state. In contrast, the morbidity index was highest in the Amazon and Tapajós, which also directly influenced the highest lethality rates. Despite the weaknesses of surveillance in each region and local vulnerability factors, such as large geographic extent, these regions presented more comorbidities and, consequently, higher mortality.

Thus, the pandemic exposed the vulnerabilities of the Unified Health System (SUS) and the surveillance system, such as inequalities in workforce distribution, infrastructure, territorial coverage, and limited capacity for diagnostic testing. From the detection of early signals of the emerging disease, the Ministry of Health (MS) implemented measures and strategies to address COVID-19, recognizing the difficulties and heterogeneity across Brazilian territories, including actions such as the preparation of a contingency plan, information sharing with the population and the press to reduce misinformation, and the publication of epidemiological bulletins on cases and deaths (8).

These factors, together with the specific characteristics of each region, are of paramount importance for the investigation and analysis of information related to these mesoregions to support the evaluation of health services. Although previous studies have described social inequalities in the Amazon, there are still no simultaneous comparative analyses of epidemiological and operational indicators stratified by mesoregions.

This study seeks to fill this gap by describing and comparing, using an ecological approach, the COVID-19 surveillance indicators in the different mesoregions of the state of Pará, incorporating sensitivity analyses, completeness assessment, and age standardization—methodological aspects frequently absent in similar analyses. The present study is relevant, as it reinforces the need to collect COVID-19 data, enabling managers and teams to clearly identify territorial challenges and design more targeted interventions for health promotion, treatment, and control of diseases, addressing the following question: How are the epidemiological and operational indicators of COVID-19 distributed in the state of Pará by health mesoregion?

## Methodology

### Design of study

This was a cross-sectional, analytical, and ecological epidemiological study based on a secondary data. Data were obtained from the Epidemiological Surveillance of Severe Acute Respiratory Syndrome through the Influenza Epidemiological Surveillance System (SIVEP-Gripe). This study followed the recommendations of *The Strengthening the Reporting of Observational Studies in Epidemiology* (STROBE) Statement: guidelines for reporting observational studies (9).

### Study location and population

**Figure 1:**
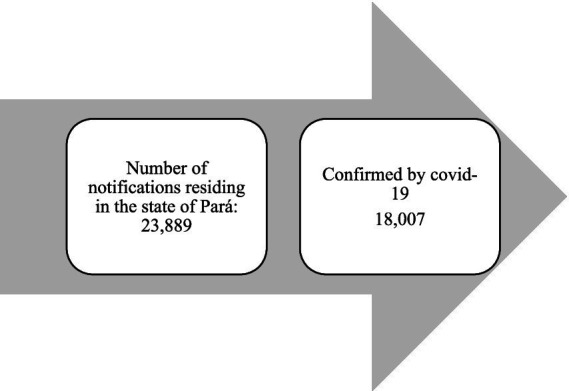
Hospitalized cases reported in the SIVEP-Gripe system in the state of Pará during the second year of the COVID-19 pandemic (2021). Source: SIVEP-GRIPE, Ministry of Health, 2023.

The study included data from the state of Pará, in the northern region of Brazil, the second largest Brazilian state, with a land area of 1,245,870.798 km^2^. The state has six mesoregions, composed of 22 microregions, with a total of 144 municipalities, and Belém as the capital (Figure 1). The territory of the state of Pará includes the Amazon Forest, the largest tropical forest in the world, and is characterized by predominantly low and flat relief, with 58% of the territory below 200 m, while altitudes above 500 m are observed in the Serra dos Carajás, Serra do Cachimbo, and Serra do Acari. The estimated population for 2020 was 8,690,745 inhabitants, with a Human Development Index (HDI) of 0.646 (10).

All hospitalized cases of severe acute respiratory infection (SARI) due to COVID-19 among residents of Pará with completed investigations between 1 January 2021 and 31 December 2021 were included. Duplicate records were excluded, retaining the first notification.

For inclusion in the epidemiological and operational comparisons, and for the outcomes of death, Intensive Care Unit (ICU) admission, and risk factors, only those residing in the state of Pará who met the diagnostic criteria, and had final classification and documented case evolution, were included, as these represent investigated and closed cases. Exclusion criteria included duplicate cases, with only the first notification retained.

All COVID-19 cases that were investigated and closed were included, as illustrated in the participant selection flowchart.

### Data collection and analysis

The variables included in the study were as follows: mesoregion of residence, risk factors, ICU admission, date of notification, date of sample collection, date of result, case closure criterion, evolution (recovery or death), date of evolution, date of hospitalization, date of investigation, and date of closure, as defined in the SIVEP-Gripe investigation form (11).

The data were made available by the State Secretariat for Public Health (SESPA) in August 2023, which is considered an acceptable period to allow completion of investigations from previous years, and were provided in Excel format.

The data were organized in a Microsoft Excel spreadsheet, and the analysis was performed using the Statistical Package for the Social Sciences (SPSS version 20; https://www.ibm.com/analytics/spss-statistics-software). Epidemiological indicators—incidence, lethality, and mortality—were calculated. The adherence tests were performed to verify the differences in the expected proportions of the epidemiological indicators by health mesoregion. Thematic maps were also generated to display indicators by health mesoregion. To improve comparability between mesoregions with distinct age structures, direct age standardization was performed using the IBGE standard population.

The cartographic bases were obtained from the Brazilian Institute of Geography and Statistics.^1^ The study area and the spatial distribution of cases by the mesoregion in Pará were analyzed using QGIS software^2^ and classified according to quartiles into classes, based on three indicators: incidence, lethality, and mortality.

Incidence refers to the rate of occurrence of a particular disease and is used to measure the frequency of new cases diagnosed in a population during a specific period. It is calculated as the number of new cases of a disease divided by the number of people at risk, multiplied by 100,000 (12).

$\frac{\begin{array}{l} \text{Number of}\;\mathrm{new}\;\text{cases of COVID}–19\;\\ \;\;\;\;\;\;\;\;\;\;\;\;\;\;\text{cases in the mesoregion} \end{array}}{\text{Population of the mesoregion}}\times 100.000$

Divided into the following quartile classes: quartile 1 (72.0–143.7), quartile 2 (143.7–225.2), quartile 3 (225.2–304.4), and quartile 4 (304.4–342.1). The mesoregions corresponding to the four quartiles were labeled on the map.

Lethality measures the severity of a disease and is defined as the proportion of deaths among individuals affected by a specific disease during a given period. The percentage is calculated as the number of deaths from a given disease in a given period divided by the number of patients with the disease in the same period, multiplied by 100 (12).

$\frac{\text{Number of COVID}‐19\;\text{deaths in the mesoregion}}{\text{Number of COVID}‐19\;\text{patients in the mesoregion}}\times 100$

Divided into the following quartile classes: quartile 1 (23.8–28.2), quartile 2 (28.2–31.1), quartile 3 (31.1–33.4), and quartile 4 (33.4–45.7). The mesoregions corresponding to the four quartiles were labeled on the map.

Mortality assessment refers to the intensity with which mortality acts on a given population. It can be considered the overall mortality rate. It is calculated by dividing the total number of deaths in a given period by the population (12).

$\frac{\text{Number of COVID}‐19\;\text{deaths in the mesoregion}}{\text{Populations of the mesoregion}}\times 1.000$

Divided into the following quartile classes: quartile 1 (22.7–46.1), quartile 2 (46.1–73.5), quartile 3 (73.5–91.1), and quartile 4 (91.1–107.8). The mesoregions corresponding to the four quartiles were labeled on the map.

To evaluate operational indicators of COVID-19 surveillance by health mesoregion, the following indicators were compared: timely notification, defined as notification of a confirmed case within 24 h (target: 80%); sample collection (target: 100%); completion of the sample collection date (target: 100%); completion of the real-time polymerase chain reaction (RT-PCR) result date (target: 100%); completion of the PCR test date (target: 100%); completion of ICU admission (target: 100%); completion of confirmation criteria (target: 100%); completion of case evolution (target: 100%); completion of the date of evolution (target: 100%); and timely case closure, defined as closure within 60 days of notification (target: 80%) (13, 14).

### Statistical analysis

To assess epidemiological indicators, incidence and mortality rates were age-standardized using the 2020 IBGE standard population. The case fatality rate was calculated as the proportion of deaths among confirmed cases.

To assess factors associated with the outcomes (death, ICU admission, and presence of risk factors), multilevel logistic regression models (mixed effects) were used to account for the hierarchical structure of the data (individuals nested within mesoregions). Variable selection was based on theoretical relevance and availability in the SIVEP-Gripe database.

For operational indicators, missing data were categorized as “not filled in.” A sensitivity analysis was performed excluding records with more than 50% missing data in critical operational variables, and the results were consistent. Multiple imputation was not performed due to the non-random pattern of the missingness, which is discussed as a limitation.

Three multivariate binary logistic regression models were fitted: the first with death as the dependent variable, the second with ICU hospitalization, and the third with the presence of risk factors. The independent variable in all three models was health mesoregion to assess associations between the dependent variables and mesoregional distribution. The alpha level of significance was set at <0.05 for all analyses. The intention was not to infer individual-level causality, but rather to examine associations at the mesoregional level, as the unit of analysis was the mesoregion.

### Ethical aspects

To comply with ethical requirements, this study followed Resolution No. 466, of 12 December 2012, which establishes the principles of bioethics, emphasizing respect for human dignity, freedom, and autonomy, as well as non-maleficence, beneficence, justice, and equity, with the objective of guaranteeing the rights and duties of all individuals involved in the research. The project was approved on 16 November 2020 by the Research Ethics Committee of the Marco School Health Center, State University of Pará (UEPA), under Approval No. 4.399.970. This study is part of an umbrella project entitled “Socio-epidemiological and spatial analysis of COVID-19 in the state of Pará during the pandemic,” conducted in partnership with the graduate program in Parasite Biology in the Amazon, State University of Pará, and the Evandro Chagas Institute (UEPA-IEC), which are responsible for the main project.

## Results

**Figure 2:**
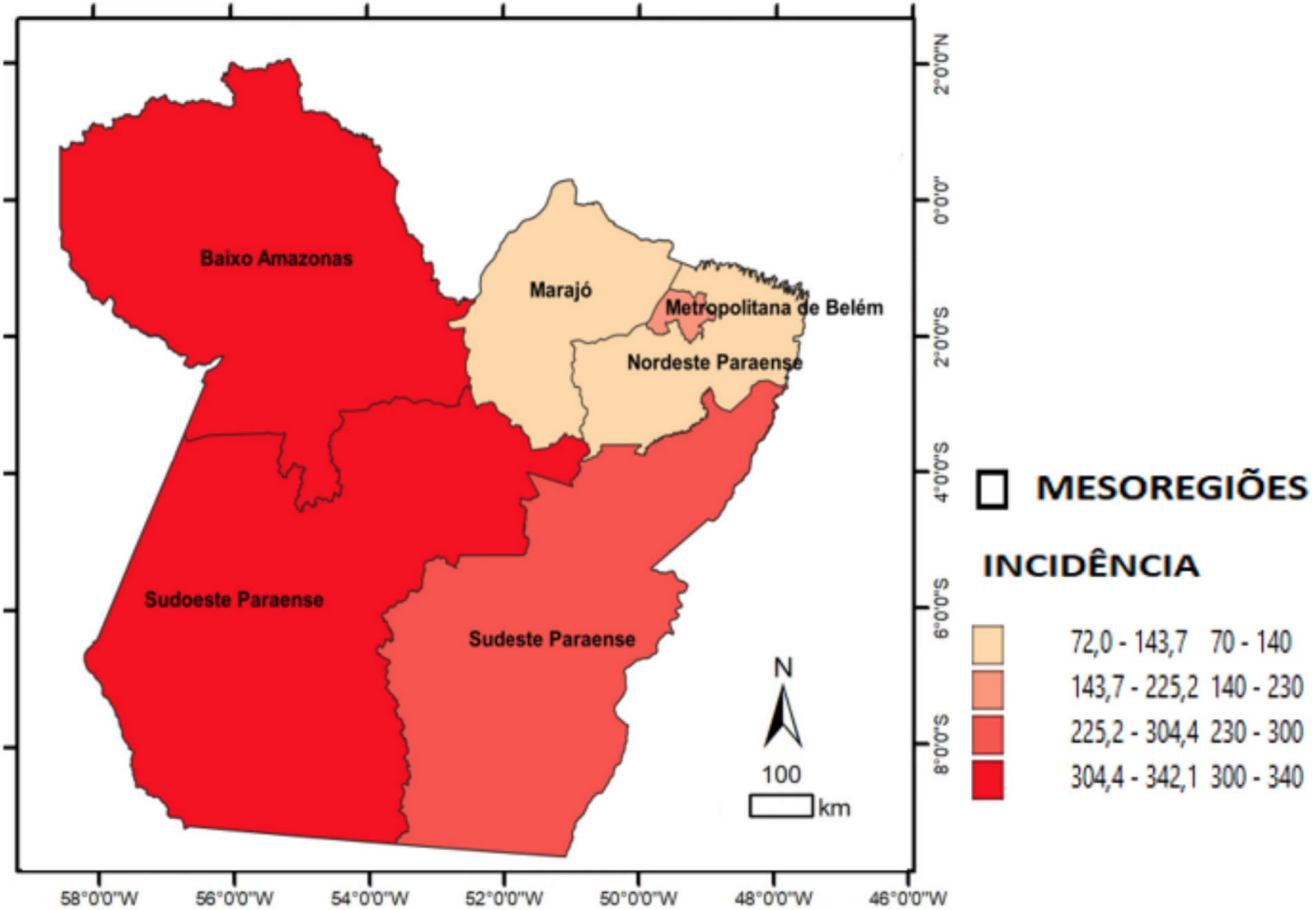
Spatial distribution of COVID-19 incidence among hospitalized patients by health mesoregion in the state of Pará, 2021. Source: IBGE, 2021. Software: QGIS. (www.qgis.org), OpenDataSUS, Ministry of Health.

**Figure 3:**
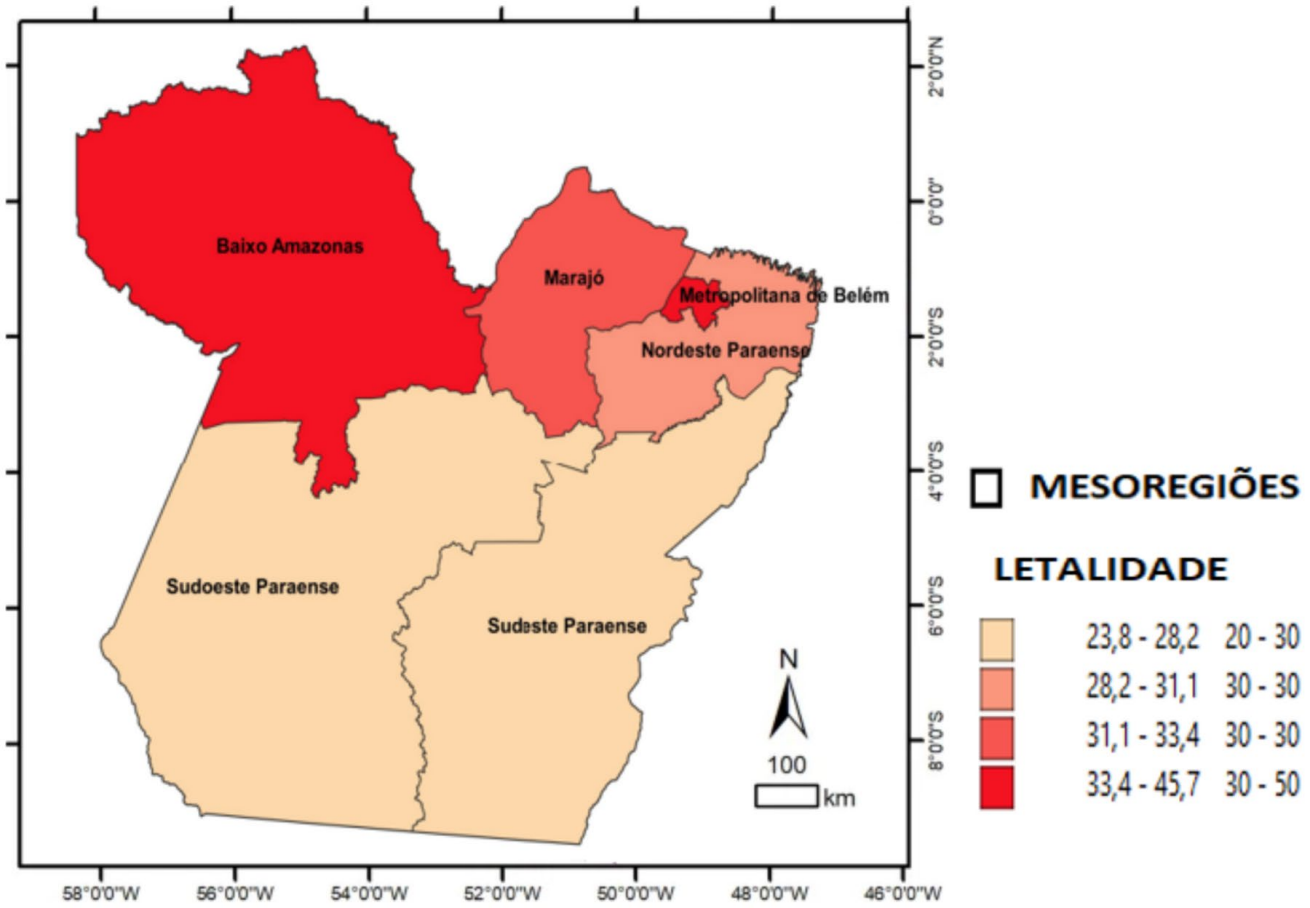
Spatial distribution of COVID-19 lethality among hospitalized patients by health mesoregion in the state of Pará, 2021. Source: IBGE, 2021. Software: QGIS. (www.qgis.org), OpenDataSUS, Ministry of Health.

**Figure 4:**
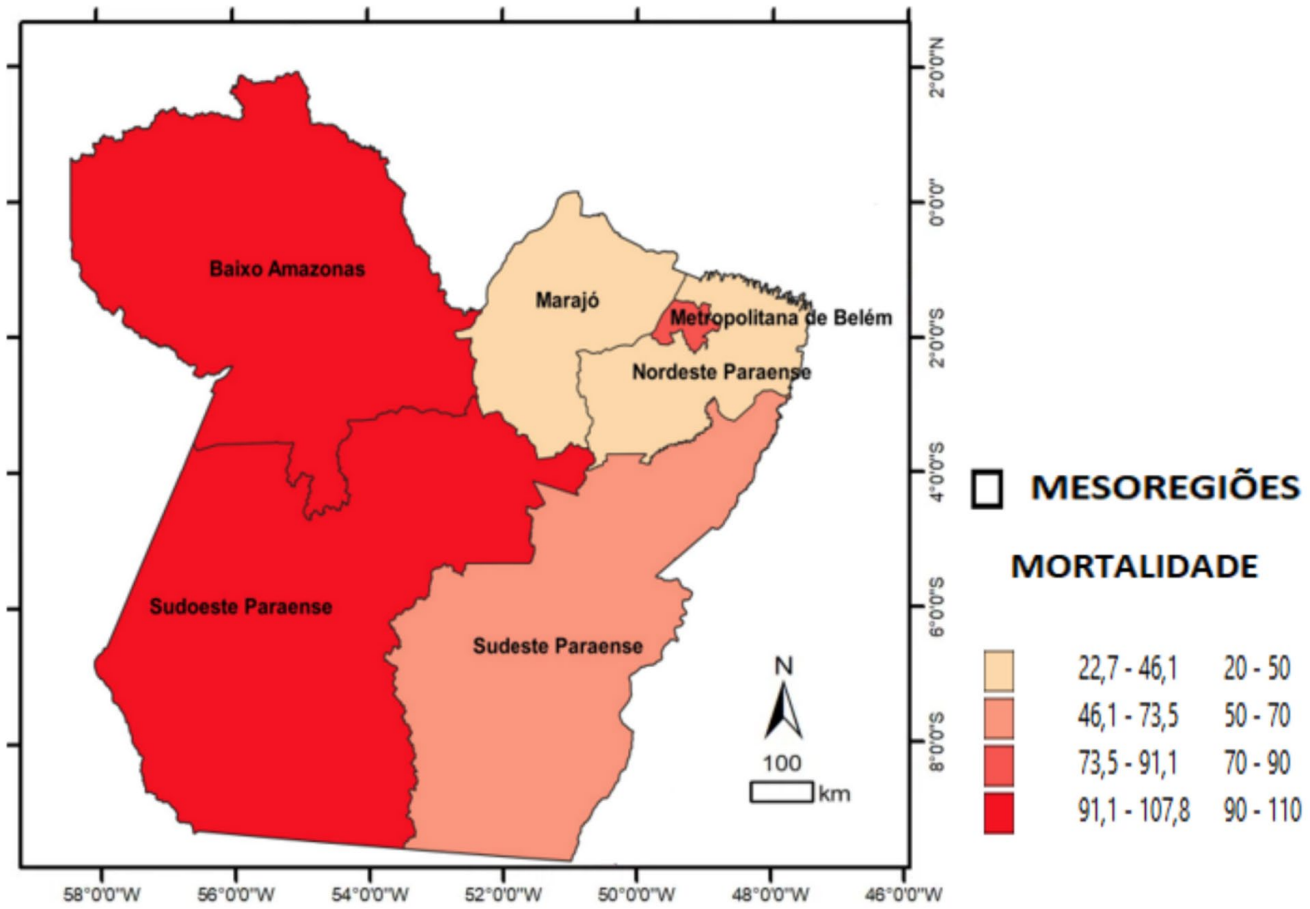
Spatial distribution of COVID-19 mortality among hospitalized patients by health mesoregion in the state of Pará, 2021. Source: IBGE, 2021. Software: QGIS. (www.qgis.org), OpenDataSUS, Ministry of Health.

The study examined reported and confirmed hospitalized COVID-19 cases. A total of 18,007 SARS surveillance notifications were included. In the comparison of epidemiological indicators, the adherence test indicated significant differences in the expected proportions across health mesoregions. In terms of incidence, significant differences were observed in the Lower Amazon, Southwest Pará, and Southeast Pará (*p* < 0.001). Regarding lethality, the highest rates were in the Lower Amazon and the Metropolitan Region of Belém (*p* < 0.001). For mortality, significant differences were observed in the Lower Amazon, the Metropolitan Region of Belém, and Southwest Pará (*p* < 0.001). The spatial distribution maps illustrate these indicators by mesoregion and highlight the areas with statistically significant differences. Overall, the mesoregions most affected by COVID-19 in terms of new cases and deaths per 100,000 inhabitants were Baixo Amazonas, Metropolitana de Belém, and Southwest do Pará (Table 1; Figures 2–4).

**Table 1:** Epidemiological indicators of COVID-19 among hospitalized patients by health mesoregion in the state of Pará, 2021.

Mesoregion	Incidence	*p*-Value	Lethality	*p*-Value	Mortality	*p*-Value
Baixo Amazonas	316.5		34		107.8	
Marajó	72		31.5		22.7	
Metropolitan of Belém	182	*<0.001	45.7	*<0.001	83.1	*<0.001
Northeast of Pará	130.9		30.7		40.2	
Southwest of Pará	342.1		27.4		93.8	
Southeast of Pará	268.3		23.8		63.9	

Source: SIVEP-Gripe, SESPA, 2021.*Adhesion test.

Operational indicators reflect the quality of epidemiological surveillance. For timely notification (target: 80%), no mesoregion achieved the target: Lower Amazon (69.9%), Marajó (43.8%), Metropolitan Region of Belém (50.7%), Northeast Pará (60.9%), Southeast Pará (67.9%), Southwest Pará (71.6%), and the state overall (62.2%). For sample collection (target: 100%), none of the mesoregions reached the target. The highest proportion was observed in Southeast Pará (92.4%), followed by Metropolitan Region of Belém (80.1%), Southwest Pará (77.3%), Lower Amazon (74.5%), Northeast Pará (73.7%), Marajó (68.5%), and the state overall (81.5%). Regarding completion of the sample collection date (target: 100%), performance remained below target across all mesoregions: Lower Amazon (74.5%), Marajó (68.5%), Metropolitan Region of Belém (80.1%), Northeast Pará (73.7%), Southeast Pará (92.4%), Southwest Pará (77.3%), and the state overall (81.5%). For completion of the PCR result date (target: 100%), none of the mesoregions reached the target: Lower Amazon (35.1%), Marajó (24.0%), Metropolitan Region of Belém (54.5%), Northeast Pará (21.6%), Southeast Pará (30.4%), Southwest Pará (14.3%), and the state overall (34.7%) (Table 2).

**Table 2:** Operational indicators for epidemiological surveillance of patients hospitalized with COVID-19 in the state of Pará, by health mesoregion, in the second year of the pandemic, 2021.

Indicators	Baixo Amazonas	Marajó	Metropolitan of Belém	Northeast of Paraense	Southeast of Paraense	Southwest of Paraense	Total	*p*-Value
Timely notification								<0.001
No	661	180	2,146	940	1,631	439	5,997	
	25.3%	43.3%	42.9%	34.9%	30.0%	23.9%	33.3%	
Unfilled	125	54	324	114	115	83	815	
	4.8%	13.0%	6.5%	4.2%	2.1%	4.5%	4.5%	
Yes	1,825	182	2,537	1,641	3,693	1,317	11,195	
	69.9%	43.8%	50.7%	60.9%	67.9%	71.6%	62.2%	
Sample collection								<0.001
No	427	50	672	427	205	211	1992	
	16.4%	12.0%	13.4%	15.8%	3.8%	11.5%	11.1%	
Unfilled	239	81	324	281	210	207	1,342	
	9.2%	19.5%	6.5%	10.4%	3.9%	11.3%	7.5%	
Yes	1945	285	4,011	1987	5,024	1,421	14,673	
	74.5%	68.5%	80.1%	73.7%	92.4%	77.3%	81.5%	
Date of collection								<0.001
Unfilled	666	131	996	708	415	418	3,334	
	25.5%	31.5%	19.9%	26.3%	7.6%	22.7%	18.5%	
Yes	1945	285	4,011	1987	5,024	1,421	14,673	
	74.5%	68.5%	80.1%	73.7%	92.4%	77.3%	81.5%	
PCR result								<0.001
Unfilled	963	143	1,045	764	1,462	540	4,917	
	36.9%	34.4%	20.9%	28.3%	26.9%	29.4%	27.3%	
Yes	1,648	273	3,962	1931	3,977	1,299	13,090	
	63.1%	65.6%	79.1%	71.7%	73.1%	70.6%	72.7%	
PCR date								<0.001
Unfilled	1,694	316	2,280	2,113	3,787	1,576	11,766	
	64.9%	76.0%	45.5%	78.4%	69.6%	85.7%	65.3%	
Yes	917	100	2,727	582	1,652	263	6,241	
	35.1%	24.0%	54.5%	21.6%	30.4%	14.3%	34.7%	
ICU admission								<0.001
Unfilled	634	82	446	282	304	258	2006	
	24.3%	19.7%	8.9%	10.5%	5.6%	14.0%	11.1%	
Yes	1977	334	4,561	2,413	5,135	1,581	16,001	
	75.7%	80.3%	91.1%	89.5%	94.4%	86.0%	88.9%	
Confirmation criteria completed								<0.001
No	242	24	81	88	133	84	652	
	9.3%	5.8%	1.6%	3.3%	2.4%	4.6%	3.6%	
Yes	2,369	392	4,926	2,607	5,306	1,755	17,355	
	90.7%	94.2%	98.4%	96.7%	97.6%	95.4%	96.4%	
Completed evolution								<0.001
No	136	39	142	118	461	151	1,047	
	5.2%	9.4%	2.8%	4.4%	8.5%	8.2%	5.8%	
Yes	2,475	377	4,865	2,577	4,978	1,688	16,960	
	94.8%	90.6%	97.2%	95.6%	91.5%	91.8%	94.2%	
Date of evolution								<0.001
No	514	68	551	373	882	263	2,651	
	19.7%	16.3%	11.0%	13.8%	16.2%	14.3%	14.7%	
Yes	2,097	348	4,456	2,322	4,557	1,576	15,356	
	80.3%	83.7%	89.0%	86.2%	83.8%	85.7%	85.3%	
Timely closure within 60 days								<0.001
No	308	76	993	298	1,060	372	3,107	
	11.8%	18.3%	19.8%	11.1%	19.5%	20.2%	17.3%	
Yes	2,303	340	4,014	2,397	4,379	1,467	14,900	
	88.2%	81.7%	80.2%	88.9%	80.5%	79.8%	82.7%	

Source: OpenDataSUS, Ministry of Health.

Completeness of the variables is a key indicator of surveillance quality. ICU admission completion ranged from 75.7% in the Lower Amazon to 94.4% in Southeast Pará, with Marajó (80.3%), Metropolitan Region of Belém (91.1%), Northeast Pará (89.5%), Southeast Pará (94.4%), Southwest Pará (86.0%), and the state overall (88.9%). The confirmation criterion was high across mesoregions: Lower Amazon (90.7%), Marajó (94.2%), Metropolitan Region of Belém (98.4%), Northeast Pará (96.7%), Southeast Pará (97.6%), Southwest Pará (95.4%), and the state overall (96.4%). For case evolution, the completion exceeded 90% in all mesoregions: Lower Amazon (94.8%), Marajó (90.6%), Metropolitan Region of Belém (97.2%), Northeast Pará (95.6%), Southeast Pará (91.5%), Southwest Pará (91.8%), and the state overall (94.2%). Completion of the date of evolution ranged from 80.3% in the Lower Amazon to 89.0% in the Metropolitan Region of Belém, with the state overall at 85.3% (Table 2).

Timely closure (within 60 days of notification; target: 80%) was achieved in five mesoregions: Lower Amazon (88.2%), Marajó (81.7%), Metropolitan Region of Belém (80.2%), Northeast Pará (88.9%), and Southeast Pará (80.5%). Southwest Pará failed to meet the target (79.8%), while the state overall achieved 82.7% (Table 2).

In the analysis of odds ratios by health mesoregion, with death as the dependent variable, the Metropolitan Region of Belém (*p* < 0.000; OR 1.896; 95% CI: 1.717–2.093) presented the highest risk, followed by the Lower Amazon (*p* < 0.010; OR 1.164; 95% CI: 1.038–1.306). In contrast, Southeast Pará (*p* < 0.000; OR 0.705; 95% CI: 0.636–0.782) and Southwest Pará (*p* < 0.016; OR 0.851; 95% CI: 0.747–0.971) mesoregions were associated with survival. Marajó was not statistically significant (*p* < 0.753) (Table 3). Regarding the risk of ICU admission, the Metropolitan Region of Belém (*p* < 0.000; OR 1.990; 95% CI: 1.795–2.207) was the only mesoregion associated with an increased risk of ICU admission. The remaining mesoregions were associated with a reduced likelihood of ICU admission: Southeast Pará (*p* < 0.000; OR 0.682; 95% CI: 0.611–0.761), Southwest Pará (*p* < 0.000; OR 0.424; 95% CI: 0.361–0.499), and Baixo Amazonas (*p* < 0.000; OR 0.702; 95% CI: 0.616–0.799). Marajó was not statistically significant (*p* < 0.160) (Table 3). With respect to the presence of risk factors (including comorbidities, puerperal status, immunodeficiency, or immunosuppression), the Metropolitan Region of Belém (*p* < 0.000; OR 1.489; 95% CI: 1.355–1.636), followed by Southwest Pará (*p* < 0.030; OR 1.141; 95% CI: 1.013–1.286). The remaining mesoregions were not statistically significant: Southeast Pará (*p* < 0.725), Marajó (*p* < 0.899), and Lower Amazon (*p* < 0.707) (Table 3).

**Table 3:** Binary logistic regression for risk of death, ICU admission, and presence of risk factors by health mesoregion among patients hospitalized with COVID-19 in Pará, 2021.

Health mesoregion	*p*-value	OR	CI 95%	
			Inferior	Superior
Risk of death				
Southeast of Pará	<0.001	0.705	0.636	0.782
Southwest of Pará	0.016	0.851	0.747	0.971
Baixo Amazonas	0.010	1.164	1.038	1.306
Metropolitan of Belém	<0.001	1.896	1.717	2.093
Marajó	0.753	1.036	0.830	1.295
Constant	<0.001	0.443		
Risk for ICU				
Southeast of Pará	<0.001	0.682	0.611	0.761
Southwest of Pará	<0.001	0.424	0.361	0.499
Baixo Amazonas	<0.001	0.702	0.616	0.799
Metropolitan of Belém	<0.001	1.990	1.795	2.207
Marajó	0.160	0.838	0.655	1.072
Constant	<0.001	0.343		
Risk for risk factor				
Southeast of Pará	0.725	0.983	0.896	1.079
Southwest of Pará	0.030	1.141	1.013	1.286
Baixo Amazonas	0.707	0.979	0.879	1.092
Metropolitan of Belém	<0.001	1.489	1.355	1.636
Marajó	0.899	0.987	0.801	1.215
Constant	<0.001	0.773		

Source: OpenDataSUS, Ministry of Health.

**Figure 5:**
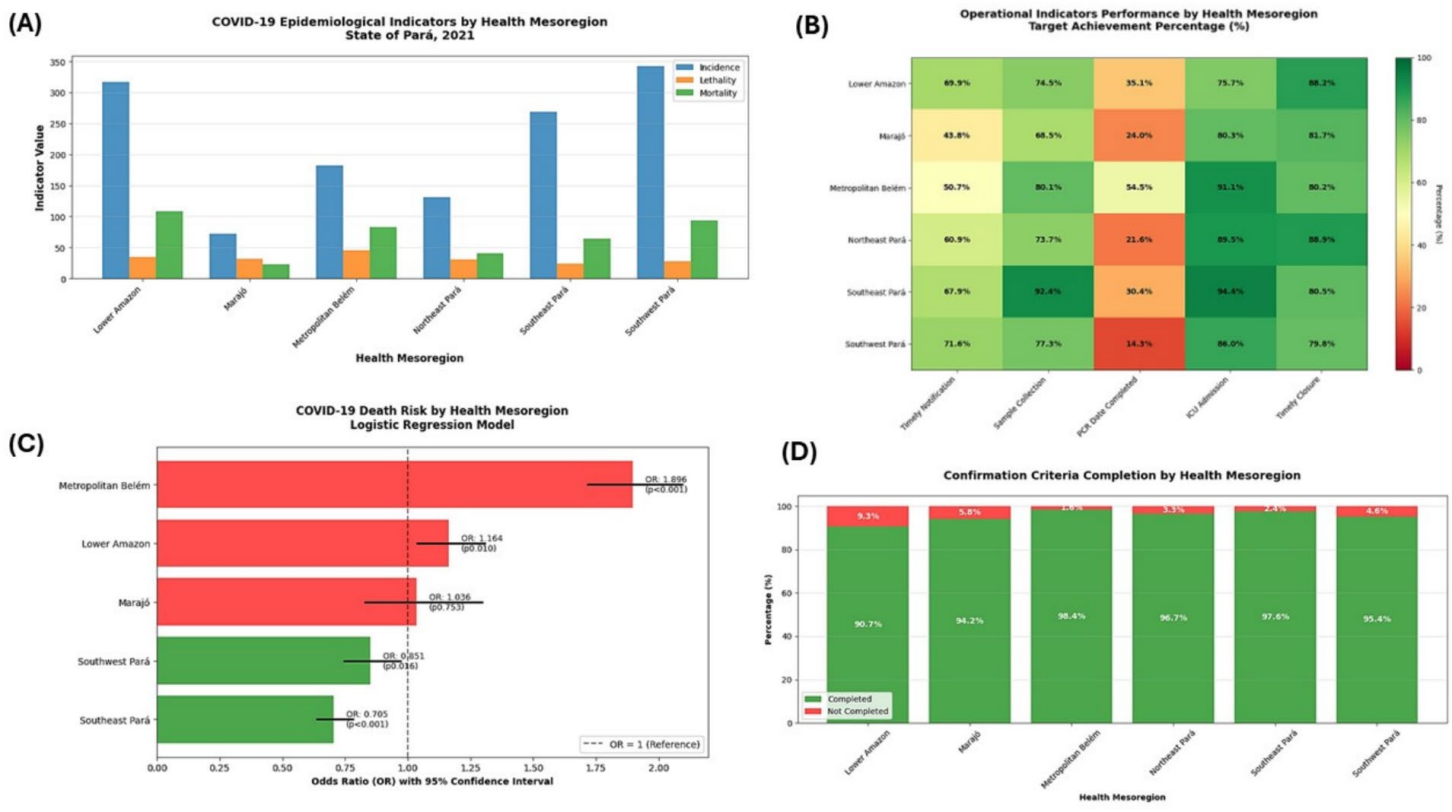
Epidemiological and operational performance of COVID-19 by health mesoregion of the state of Pará, 2021. **(A)** Epidemiological indicators of COVID-19 by health mesoregion (bar graph comparing incidence, lethality, and mortality across the seven mesoregions of Pará). **(B)** Heatmap of the performance of operational indicators by mesoregion (percentage of goal achievement). **(C)** Risk of death from COVID-19 by mesoregion—logistic regression model. **(D)** Proportion of completion of COVID-19 confirmation criteria by mesoregion.

The graphical representations of the data are presented in Figure 5. Strong territorial heterogeneity is observed, with the Lower Amazon and Southeast Pará regions showing the highest incidence rates, while mortality and case fatality rates vary unevenly across mesoregions. The heatmap illustrates the percentage of compliance with operational COVID-19 surveillance targets, including timely notification, sample collection, PCR result entry, ICU admission recording, and timely case closure, with greener shades indicating better performance. Significant differences are evident: Metropolitan Belém and Lower Amazon show higher compliance rates, while Marajó and part of Northeast Pará show lower performance, particularly for PCR entry. The Metropolitan Region of Belém presents the highest risk of death (OR 1.866; *p* < 0.001), followed by the Lower Amazon (OR 1.164; *p* = 0.010). The Southeast Pará and Southwest Pará regions show a significantly lower risk (OR < 1). Overall, the model highlights substantial territorial inequalities in COVID-19 severity. All mesoregions show high levels of completeness in disease confirmation criteria (above 90%), with slight variations: Metropolitan Belém and Southeast Pará have the best rates of mandatory completion.

## Discussion

This was the first study to evaluate the epidemiological and operational indicators of COVID-19 in the state of Pará during the COVID-19 pandemic. The large territorial extension of Pará and the differences in social determinants across health mesoregions are the main factors associated with worse COVID-19 outcomes, as timely investigation and early diagnosis enable surveillance to implement actions to control transmission and support efficient patient recovery.

In the mesoregions farthest from the metropolitan region of the state, outcomes were most affected by the distance from adequate healthcare infrastructure to carry out all stages of clinical management and optimal surveillance for patients with COVID-19, resulting in higher mortality and disparities in the quality of care and testing. This occurs because the metropolitan region is where the most complex hospitalization care and surveillance services are centralized, including the supply of gold-standard tests for COVID-19.

A study in the state of Pará in 2020 evaluated 100,819 cases. The municipalities with the highest incidence rates were Parauapebas, Canaã dos Carajás, and Jacareacanga. Similarities were observed among the mesoregions of the Lower Amazon, Marajó, and Southwest Pará in terms of the highest morbidity rates. The data showed that most cases occurred among young female adults with classic flu symptoms and chronic diseases. The data suggested that the highest incidences no longer occurred in the metropolitan region of the state. The study showed that the lethality rate was higher than that in Brazil and may be associated with the greater impact of the disease on this Amazonian population or with factors associated with weak epidemiological surveillance in the notification of recovered cases (7).

Another study described the incidence of COVID-19 and its influence on social inequalities in Ananindeua and Marituba, Pará. The study was carried out by comparing maps with social indicators of income, race, and sex and analyzing the relationship between the location of observation and hospitalization health units and subnormal clusters. The analysis highlighted recurring spatial patterns in the indicators, with overlapping negative bands in the same portions of the territory and the best indices in complementary areas, showing the socio-spatial inequality of the conurbation, which has been neglected by the policies that would provide better conditions for social isolation. The author also pointed out that the existing public healthcare structure, including basic health units (UBS) and SUS hospitals that treat COVID-19 cases, does not coincide with the poorest areas, where there are high household density, low income, and women-headed households (15).

In our study, the incidences were higher in the Lower Amazon and Southwest Pará mesoregions, which are even farther from the metropolitan area and have greater social vulnerabilities. Another study also highlighted that in Belém, PA, COVID-19 was more abundant in poorer regions, influenced by social determinants, which are risk factors for COVID-19 outcomes, and that the strategies to combat it did not consider local peculiarities, such as social determinants, making control more difficult if these factors were not addressed (16). This pattern is also observed in the other mesoregions of the state, which are even more vulnerable than the metropolitan region.

A survey highlighted COVID-19 in the state of Pará, noting that the presence of different social impacts and inequities in access to healthcare, exacerbated by the disease, makes it important to highlight the performance of government actions and individual initiatives, aiming at the implementation of public strategies that are effective in controlling the pandemic (17).

The state has a diversity of traditional peoples and a large geographical extension, which hinders access to healthcare and information. Therefore, a study in the Xingu region carried out a project for the Xingu population, especially Indigenous and rural communities, which involved publications on social networks and information on prevention measures, risk groups, and social isolation. For communities without internet access, a local radio station provided information to rural and remote Indigenous communities. The project also created a booklet, in Portuguese and Kayapó, with guidelines for the prevention of COVID-19 for the Indigenous people of the Middle Xingu (18). Strategies like this are important for health promotion education in regions with social vulnerability to control COVID-19.

Matos et al. (19) conducted a study in the state of Pará, showing the evolution and repercussions of COVID-19 after 1 year of the pandemic. There were 420,372 cases, predominantly among women (53.4%), and deaths predominantly among men (60.1%). The cases were concentrated in Belém (20.45%). Regarding age groups, young adults accounted for 23.5% of cases, while deaths were most frequent in the 70–79-year age group (26.7%). The most frequent comorbidities were heart disease (43%) and diabetes (33%), particularly among patients requiring intensive care (87.63%).

Geha et al. (20) compared the epidemiological characteristics, similarities, and differences of influenza A(H1N1) pdm09 and COVID-19 in Pará. Pará had 124,934 cases of COVID-19 and 783 cases of pandemic influenza. The 30–39-year age group accounted for 24.95% of COVID-19 cases, with higher mortality among older adults (74.00%), while the influenza A (H1N1) pdm09 virus predominantly affected children and adolescents aged 10–19 years (31.42%), with mortality highest among those aged 20–29 years (26.83%). COVID-19 showed a more dispersed distribution of cases compared with the influenza A(H1N1) pdm09 pandemic. The findings indicate that the epidemiological scenario of the influenza A(H1N1) pdm09 pandemic in Pará highlights the need for modifications in strategic planning to combat the COVID-19 pandemic, given the differences in affected population groups and clinical conditions.

A survey of 16,000 COVID-19–associated hospitalizations in the US states of California, Colorado, Connecticut, Georgia, Iowa, Maryland, Michigan, Minnesota, New Mexico, New York, Ohio, Oregon, Tennessee, and Utah showed that 34.8% occurred among non-Hispanic white people (white), 36.3% among non-Hispanic black people (black), and 18.2% among Hispanic or Latino (Hispanic) people. The age-adjusted COVID-19–associated hospitalization rate was 151.6 (95% confidence interval (CI): 147.1–156.1) in census tracts with >15.2–83.2% of people living below the federal poverty level (high-poverty census tracts) and 75.5 (95% CI: 72.9–78.1) in census tracts with 0–4.9% of people living below the federal poverty level (low-poverty census tracts). Among white, black, and Hispanic people living in high-poverty census tracts, age-adjusted hospitalization rates were 120.3 (95% CI: 112.3–128.2), 252.2 (95% CI: 241.4–263.0) and 341.1 (95% CI: 317.3–365.0), respectively, compared with 58.2 (95% CI: 55.4–61.1), 304.0 (95% CI: 282.4–325.6), and 540.3 (95% CI: 477.0–603.6), respectively, in low-poverty census tracts. Overall, COVID-19–associated hospitalization rates were highest in high-poverty census tracts, but rates among black and Hispanic people were high, regardless of poverty level. Public health professionals should ensure that mitigation measures and vaccination campaigns address the needs of racial/ethnic minority groups and people living in high-poverty census tracts (21).

Corroborating our study, higher incidences and mortality were observed in the Lower Amazon and Southwest Pará, mesoregions with greater social vulnerability than other mesoregions, as reported in the annual report of the Amazon Foundation for the Support of Studies and Research (FAPESPA) (22).

Social factors influence the severity of COVID-19; areas with lower income experience inequalities in access to healthcare, which are associated with worse COVID-19 outcomes (23). Previous research indicates that low income and race are associated with greater crowding in workplaces and living spaces, which can increase the likelihood of disease transmission (24).

A study evaluated the social determinants related to the incidence, mortality, and case fatality rate of COVID-19 in Brazil in 2020. A total of 44.8% of the municipalities reported COVID-19 cases and 14.7% reported deaths. Among municipalities with reported cases, 56.2% had very low human development (COVID-19 incidence rate: 59.00 per 100,000; mortality rate: 36.75 per 100,000), and 52.8% had very high social vulnerability (COVID-19 incidence rate: 41.68 per 100,000; mortality rate: 27.46 per 100,000). These findings highlight the need to adopt measures that account for local social factors to contain the pandemic (25).

A study conducted in the United States found that individuals living in areas with greater social vulnerabilities were two times more likely to reach ICU bed capacity than those in less vulnerable areas (OR = 2.15, 95% CI: 1.41–3.29). The link between social vulnerability and critical ICU capacity highlights underlying structural inequalities in access to healthcare and provides policymakers with an opportunity to prevent stressed ICU capacity from exacerbating COVID-19 inequalities (26). In our study, 94.4% of patients admitted to the ICU were from the southeastern region of Pará, corroborating these findings, as it is a mesoregion with high social vulnerability.

Another survey in Spain included 10,110 patients admitted to 18 Spanish hospitals (mean age 68 (IQR 54–80) years; 44.5% female; 14.8% not born in Spain). Of these, 779 (7.7%) patients were admitted to ICUs, and 1,678 (16.6%) patients died during hospitalization. Age, male sex, immigrant status, and low hospital saturation were independently associated with ICU admission. Age, male sex, immigrant status, average per capita income percentile, and hospital experience were independently associated with in-hospital mortality. Social determinants, such as living in low-income areas and being born in Latin American countries, were associated with increased odds of ICU admission and in-hospital mortality. There was considerable variation in results across Spanish centers (27).

Karmakar et al. (28), in the United States, highlighted that SARS-CoV-2 did not create conditions for health disparities or reveal previously unrecognized social inequalities. Instead, the pandemic exacerbated long-standing racial/ethnic, social, political, and economic inequalities, resulting in the most marginalized and under-resourced communities experiencing the worst outcomes. A wide range of social factors, including socioeconomic status, racial/ethnic minority status, household composition, and environmental factors, have been significantly associated with COVID-19 incidence and mortality and are considered key drivers of the racial/ethnic and social disparities observed during the COVID-19 pandemic in the United States.

To effectively reduce disparities in COVID-19 and future epidemics or pandemics, these social risk factors and their root causes must be addressed through targeted policy measures and social investment (28). Similarly, a study in Brazil in 2020 highlighted that areas in the North and Northeast regions were at high risk of COVID-19 outbreaks and were highly socially vulnerable and that these regions became the most severely affected over the course of the pandemic (29).

A survey in Minas Gerais (MG) in 2020 evaluated data quality attributes, timeliness, and representativeness of COVID-19 surveillance in the Jequitinhonha health macroregion. The study assessed SG in the e-SUS Notifica system and SARS in SIVEP-Gripe. In e-SUS Notifica, 38.38% of notifications were inconsistent, and 4.67% were duplicated; regarding case closure, disease evolution, and final classification, approximately 40% were incomplete. In SIVEP-Gripe, data quality in terms of inconsistent and duplicate notifications was considered excellent, although 14% of records were closed late and 18% were incomplete. In terms of timeliness, performance was excellent, with 82% of cases notified within 7 days and 96% entered into the system within 30 days. The authors concluded that surveillance systems require improvements in the consistency of e-SUS Notifica data and in the quality of case closure in both e-SUS Notifica and SIVEP-Gripe (30).

A hospital-based study in Rio Grande do Sul evaluated the quality of SARS notifications associated with COVID-19 in SIVEP-Gripe, aiming to identify the strengths and weaknesses of hospital epidemiological surveillance at the local level. Only 21% of notifications were timely, while overall completeness was satisfactory, except for education level, which was missing in 58.6% of records. Agreement between variables was unsatisfactory due to a high rate of inconsistencies. These findings highlight the need to improve the information system through strategies such as database integration, revision of notification forms, and training of health and administrative professionals (31).

In Aracajú, Sergipe, a study evaluated SIVEP-Gripe data on COVID-19 deaths from March 2020 to 30 June 2021. The analysis showed an average completeness of 81% for mandatory and essential variables. Timeliness was inadequate for notification (44.35%) and nasopharyngeal sample collection (69.28%) but adequate for case closure (96.75%). The authors concluded that SIVEP-Gripe requires improvement from sentinel units through to surveillance activities within health departments and that training health professionals and system operators is a strategy to enhance quality indicators (32).

A study in the United States examined surveillance of hospitalized patients with COVID-19 and concluded that COVID-19-associated hospital admission rates and percentages of positive test results, COVID-19 emergency department visits, and COVID-19 deaths are adequate and timely indicators of trends in COVID-19 activity and severity (33).

Another study in the United States examining COVID-19 surveillance highlighted changes to the national COVID-19 monitoring strategy and the COVID Data Tracker, which conducts immunization surveillance, capitalizing on marked improvements in various surveillance systems. Weekly COVID-19 hospital admission levels and the percentage of all COVID-19–associated deaths were identified as primary indicators of surveillance. Emergency department visits and the percentage of positive laboratory test results for SARS-CoV-2 were also identified as indicators that help detect early changes in trends. Genomic surveillance will continue to help identify and monitor SARS-CoV-2 variants (34).

This is the first study to evaluate both epidemiological and operational indicators of COVID-19 surveillance across health mesoregions in the state of Pará. Our findings show significant disparities across both indicators, with the most vulnerable and remote mesoregions (Lower Amazon, Southwest Pará, and Southeast Pará) experiencing the worst outcomes. These mesoregions are farther from Belém’s metropolitan area, where health services are concentrated. However, we acknowledge that unmeasured confounding (e.g., comorbidities, health service capacity) may partially explain the association. The operational indicators reveal weaknesses in the surveillance system, particularly in timely notification and completion of laboratory data. These findings can inform targeted interventions to improve surveillance in the most affected mesoregions. Research highlights that syndromic surveillance of flu-like symptoms is an essential tool for identifying increases in cases and clusters, which can help predict increases in hospitalizations in the community and reduce severe cases and deaths from COVID-19 (35).

The limitations of the study and the risk of bias arise because the data are secondary from epidemiological surveillance data, and the completion of the variables depends on health professionals. The lack of understanding of the relevance of mandatory notifications by health professionals directly impacts epidemiology, thereby contributing to underreporting, as the information collected serves as a reference for social and collective health issues. The ecological design prevents causal inferences. We could not adjust for all potential confounders, such as comorbidities, access to testing, and health service capacity.

The data are subject to underreporting and missing information, which may be more pronounced in the most vulnerable mesoregions. We handled missing data by sensitivity analysis, but the pattern of missingness may bias the results. The use of multilevel models is a strength, but residual confounding remains. In addition, we highlight that fragility in regional surveillance may mask a more unfavorable situation in the region, as mesoregions farther away and with greater social vulnerabilities tend to experience higher underreporting due to a lack of human, physical, and technological resources.

## Conclusion

In this study, we examine the differences in epidemiological and operational indicators of COVID-19 in the state of Pará. We highlight differences across health mesoregions in both epidemiological and operational indicators. The most affected mesoregions were the Lower Amazon, Southwest Pará, and Southeast Pará, which are precisely those with high social vulnerabilities and are farther from the metropolitan area of Belém, where health services with better hospital and laboratory structures are concentrated.

Thus, social inequality leads to limited access to healthcare and disparities in COVID-19 outcomes, both in epidemiology and in the quality of surveillance. COVID-19 did not generate social inequality, but rather exacerbated existing social vulnerabilities in these mesoregions. Therefore, control measures and strategies to combat the COVID-19 pandemic must consider social factors, which municipal, state, and federal managers must address, as measures that incorporate social factors are more effective in reducing cases, hospitalizations, and deaths, and consequently in improving quality of life, health promotion, and reducing costs to the Unified Health System. As a strategy and an important priority, vaccination is provided by the health mesoregions; however, the large territorial extension and the rural and Indigenous populations pose challenges to achieving optimal coverage. Therefore, the SUS must prioritize and guarantee vaccines for all traditional peoples and for socioeconomically disadvantaged populations.

The COVID-19 pandemic has strengthened respiratory virus surveillance in Brazil. These data may serve as lessons for the improvement of SARS surveillance and data quality, with training and the provision of adequate infrastructure for both SG sentinel surveillance to monitor the circulation of respiratory viruses and hospital surveillance, which serves as an indicator through bed occupancy and gold-standard tests for diagnosing etiologies, thereby enabling better early epidemiological management in regions with high social vulnerability.

## Data Availability

The original contributions presented in the study are included in the article/supplementary material, further inquiries can be directed to the corresponding author/s.

## Footnotes

1
http://www.ibge.gov.br/

2
https://qgis.org/pt_BR/site/
